# Ethyl 2-[6-(4-methyl­benzo­yl)-7-phenyl-2,3-di­hydro-1*H*-pyrrolizin-5-yl]-2-oxo­acetate

**DOI:** 10.1107/S1600536813029401

**Published:** 2013-11-06

**Authors:** Jia-liang Zhong, Wen-xia Sun, Fu-li Zhang, Li-hong Liu, He Liu

**Affiliations:** a Shanghai Institute of Pharmaceutical Industry, Shanghai 200040, China; b Beijing Chao-Yang Hospital Affiliated with Beijing Capital, Medical University, Beijing 100020, China

## Abstract

In the title compound, C_25_H_23_NO_4_, the pyrrolizine ring is approximately planar with an r.m.s deviation from planarity of 0.0053 Å, while the fused di­hydro­pyrrolizine ring adopts an envelope conformation with the C atom connected to two CH_2_ as the flap. The dihedral angles between the fused ring system and the phenyl and methyl­benzoyl rings are 41.65 (11) and 66.30 (8)°, respectively. In the crystal, weak C—H⋯O hydrogen bonds and C—H⋯π inter­actions occur. One mol­ecule is linked to five adjacent ones through eight hydrogen bonds, forming a three-dimensional network.

## Related literature

For the synthesis of the title compound, see: Itoh *et al.* (1984 ▶). For similar structures, see: Liu *et al.* (2007 ▶, 2013 ▶).
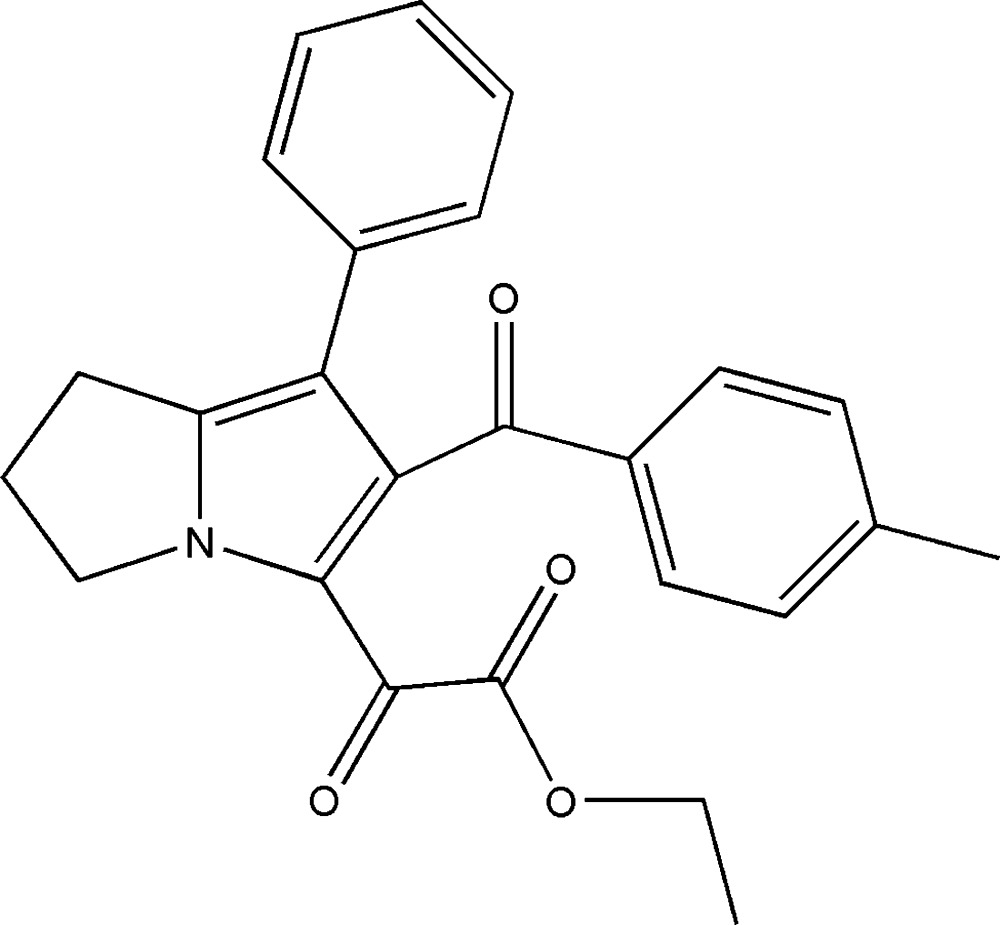


## Experimental

### 

#### Crystal data

C_25_H_23_NO_4_
*M* *_r_* = 401.44
Monoclinic, 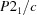

*a* = 8.8949 (18) Å
*b* = 9.0003 (18) Å
*c* = 25.580 (5) Å
β = 96.75 (3)°
*V* = 2033.7 (7) Å^3^
*Z* = 4
Mo *K*α radiation
μ = 0.09 mm^−1^
*T* = 296 K
0.26 × 0.15 × 0.10 mm

#### Data collection

Bruker APEXII CCD diffractometer
16259 measured reflections
3775 independent reflections
1930 reflections with *I* > 2σ(*I*)
*R*_int_ = 0.070

#### Refinement

*R*[*F* ^2^ > 2σ(*F* ^2^)] = 0.062
*wR*(*F* ^2^) = 0.194
*S* = 0.97
3775 reflections
274 parameters
H-atom parameters constrained
Δρ_max_ = 0.16 e Å^−3^
Δρ_min_ = −0.36 e Å^−3^

### 

Data collection: *APEX2* (Bruker, 2009 ▶); cell refinement: *SAINT* (Bruker, 2009 ▶); data reduction: *SAINT*; program(s) used to solve structure: *SHELXS97* (Sheldrick, 2008 ▶); program(s) used to refine structure: *SHELXL97* (Sheldrick, 2008 ▶); molecular graphics: *SHELXTL* (Sheldrick, 2008 ▶); software used to prepare material for publication: *SHELXTL* and *PLATON* (Spek, 2009 ▶).

## Supplementary Material

Crystal structure: contains datablock(s) I, global. DOI: 10.1107/S1600536813029401/zp2010sup1.cif

Structure factors: contains datablock(s) I. DOI: 10.1107/S1600536813029401/zp2010Isup2.hkl

Additional supplementary materials:  crystallographic information; 3D view; checkCIF report

## Figures and Tables

**Table 1:** Hydrogen-bond geometry (Å, °) *Cg*4 is the centroid of the C21–C26 phenyl ring.

*D*—H⋯*A*	*D*—H	H⋯*A*	*D*⋯*A*	*D*—H⋯*A*
C20—H20*B*⋯O3^i^	0.96	2.64	3.268 (6)	123
C12—H12*B*⋯O2^ii^	0.96	2.63	3.579 (5)	170
C12—H12*A*⋯O1^iii^	0.96	2.66	3.376 (5)	132
C2—H2*A*⋯O4^iv^	0.97	2.70	3.391 (5)	128
C19—H19⋯*Cg*4^v^	0.93	2.96	3.769 (5)	147

Symmetry codes: (i) 
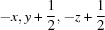
; (ii) 
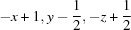
; (iii) 
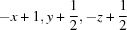
; (iv) 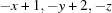; (v) 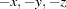.

**Fig. 1.**
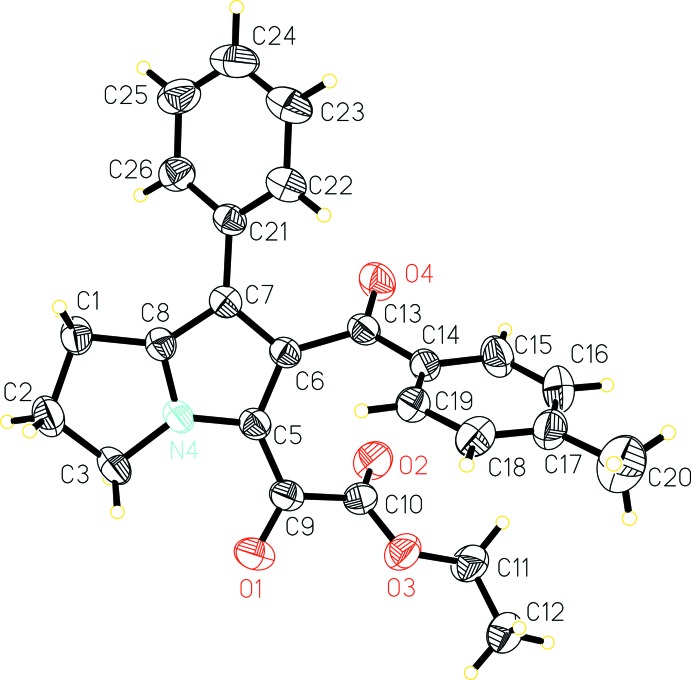
View of the molecule of (I) showing the atom-labelling scheme. Displacement ellipsoids are drawn at the 30% probability level.

**Fig. 2.**
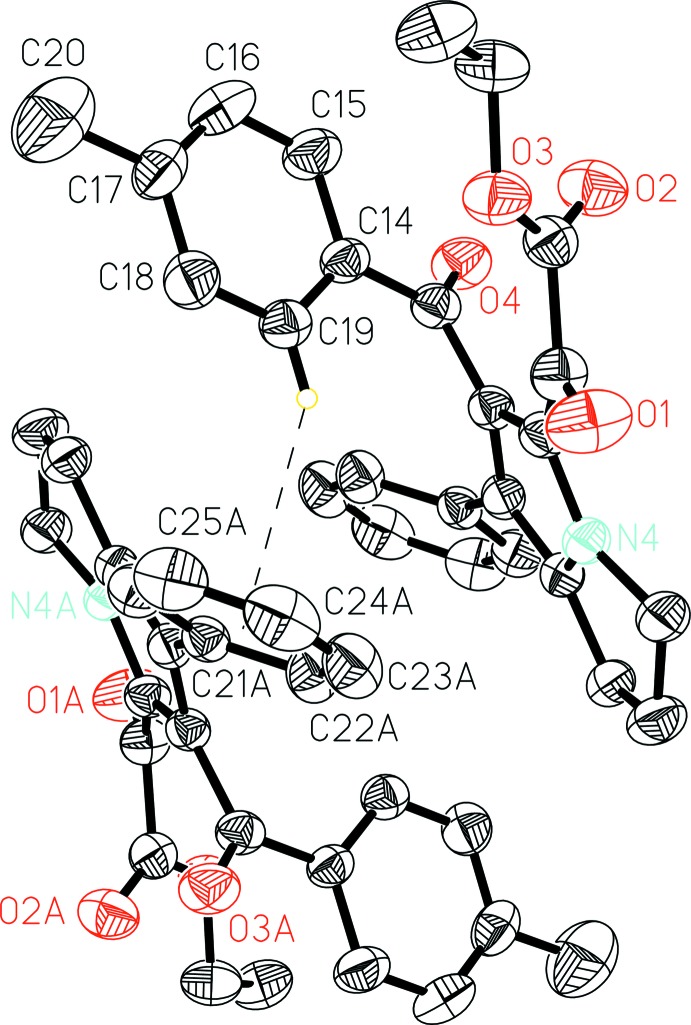
The C—H···π interaction, dashed lines. Non-essential H atoms are omitted for clarity. Symmetry code: (i) -x, -y, -z.

## supplementary crystallographic information

### 1. Comment

The title compound, (I) (Fig. 1), ethyl 2-(6-(4-methylbenzoyl)-7-phenyl -2,3-dihydro-1*H*-pyrrolizin-5-yl)-2-oxoacetate was synthesized from 6-(4-Methylbenzoyl)-7-phenyl-2,3-dihydro-pyrrolizine and ethyl oxalyl monochloride according to a general literature procedure of Itoh *et al.*, (1984).

The title compound was an analogue of C_20_H_19_NOS (Liu, *et al.*, 2013). The pyrrolizine ring is almost planar, while the fused dihydro-pyrrolizine ring adopts an envelope conformation. The dihedral angle between ring A (C1/C2/C3/N4/C5/C6/C7/C8) and phenyl ring B (C21—C26) is 41.65 (11)°, the dihedral angle between ring A and ring C (C13/C14/C15/C15/C17/C18/C19/C20) is 66.30 (8)°, and the dihedral angle between ring B and ring C is 74.31 (11)°. The torsion angle of O1/C9/C10/O2 was 131.5 (4)°. As a result, three side chains of ring A arranged themselves like propeller due to steric.

In the crystal, a series of weak intermolecular C—H···O (Table 1) hydrogen bonds can be found. Compared to C_20_H_19_NOS (Liu, *et al.*, 2013), three O atoms of ethyloxalyl form three H-bonds, which help stablizing the crysatl packing. One molecule link to five adjacent ones through eight H-bonds to form three-dimensional infinite packing. An analysis by *PLATON* (Spek, 2009) shows C19—H19···π interaction with H19 to ring B centroid(*Cg*4) distance of 2.96 Å(Table 1, Fig2).

### 2. Experimental

A stirred solution of 6-(4-Methylbenzoyl)-7-phenyl-2,3-dihydro- pyrrolizine in anhydrous CH_2_Cl_2_ was treated with AlCl_3_. A solution of ethyl oxalyl monochloride in anhydrous CH_2_Cl_2_ was added. The mixture was stirred for 4 h at room temperature, and cooled to 273 K, an aqueous solution of HCl(w/w10%) was then added and the resulting solution was stirred for 1 h. After addition of water to form a clear aqueous layer, the organic layer was separated and dried(anhydrous Na_2_SO_4_). Then the solution was evaporated under reduced pressure and purified by chromatography on silica gel column, eluting with a petroleum ether/acetone mixture (2:1) to give 65% yield of light yellow solid. The purity of the title compound was verified by elemental analysis: calculated for C_25_H_23_NO_4_: C 74.80, H 5.77, N 3.49; found C 65.72, H 4.39, N 3.83. EI—MS m/*z*: 402 (*M*+H)^+^.

The crystal appropriate for X-ray data collection was obtained from acetone solution at room temperature after two days.

### 3. Refinement

All H atoms were placed in geometically idealized positions and constrained to ride on their parent atoms with C—H distances of 0.93(0.97 for CH_2_)Å for CH, and *U*_iso_(H) = 1.2(1.5 for CH3)*U*_eq_(C). Four H atoms taking part in the hydrogen-bonds can be found on the difference Fourier maps although the position of H20B was not perfect.

### Crystal data

**Table:**

C_25_H_23_NO_4_	*F*(000) = 848
*M**_r_* = 401.44	*D*_x_ = 1.311 Mg m^−^^3^
Monoclinic, *P*2_1_/*c*	Mo *K*α radiation, λ = 0.71073 Å
Hall symbol: -P 2ybc	Cell parameters from 9483 reflections
*a* = 8.8949 (18) Å	θ = 3.1–27.5°
*b* = 9.0003 (18) Å	µ = 0.09 mm^−^^1^
*c* = 25.580 (5) Å	*T* = 296 K
β = 96.75 (3)°	Needle, yellow
*V* = 2033.7 (7) Å^3^	0.26 × 0.15 × 0.10 mm
*Z* = 4	

### Data collection

**Table:**

Bruker APEXII CCD diffractometer	1930 reflections with *I* > 2σ(*I*)
Radiation source: fine-focus sealed tube	*R*_int_ = 0.070
Graphite monochromator	θ_max_ = 25.5°, θ_min_ = 3.2°
φ and ω scans	*h* = −10→10
16259 measured reflections	*k* = −10→10
3775 independent reflections	*l* = −30→30

### Refinement

**Table:**

Refinement on *F*^2^	Secondary atom site location: difference Fourier map
Least-squares matrix: full	Hydrogen site location: inferred from neighbouring sites
*R*[*F*^2^ > 2σ(*F*^2^)] = 0.062	H-atom parameters constrained
*wR*(*F*^2^) = 0.194	*w* = 1/[σ^2^(*F*_o_^2^) + (0.1076*P*)^2^] where *P* = (*F*_o_^2^ + 2*F*_c_^2^)/3
*S* = 0.97	(Δ/σ)_max_ < 0.001
3775 reflections	Δρ_max_ = 0.16 e Å^−^^3^
274 parameters	Δρ_min_ = −0.36 e Å^−^^3^
0 restraints	Extinction correction: *SHELXL*, Fc^*^=kFc[1+0.001xFc^2^λ^3^/sin(2θ)]^-1/4^
Primary atom site location: structure-invariant direct methods	Extinction coefficient: 0.007 (2)

### Special details

**Table:**

Geometry. All e.s.d.'s (except the e.s.d. in the dihedral angle between two l.s. planes) are estimated using the full covariance matrix. The cell e.s.d.'s are taken into account individually in the estimation of e.s.d.'s in distances, angles and torsion angles; correlations between e.s.d.'s in cell parameters are only used when they are defined by crystal symmetry. An approximate (isotropic) treatment of cell e.s.d.'s is used for estimating e.s.d.'s involving l.s. planes.
Refinement. Refinement of *F*^2^ against ALL reflections. The weighted *R*-factor *wR* and goodness of fit *S* are based on *F*^2^, conventional *R*-factors *R* are based on *F*, with *F* set to zero for negative *F*^2^. The threshold expression of *F*^2^ > σ(*F*^2^) is used only for calculating *R*-factors(gt) *etc*. and is not relevant to the choice of reflections for refinement. *R*-factors based on *F*^2^ are statistically about twice as large as those based on *F*, and *R*- factors based on ALL data will be even larger.

### Fractional atomic coordinates and isotropic or equivalent isotropic displacement parameters (Å^2^)

**Table:**

	*x*	*y*	*z*	*U*_iso_*/*U*_eq_	
O1	0.3855 (4)	0.7209 (3)	0.14060 (9)	0.1067 (10)	
O2	0.5168 (3)	1.0611 (3)	0.16709 (9)	0.0918 (8)	
O3	0.3842 (3)	0.9266 (3)	0.21838 (8)	0.0818 (7)	
O4	0.3274 (3)	1.2999 (3)	0.07415 (9)	0.0829 (7)	
N4	0.3342 (3)	0.8148 (3)	0.03486 (9)	0.0588 (7)	
C1	0.2962 (4)	0.7905 (4)	−0.05606 (12)	0.0695 (9)	
H1A	0.3753	0.8216	−0.0768	0.083*	
H1B	0.2001	0.7897	−0.0783	0.083*	
C2	0.3308 (4)	0.6400 (4)	−0.03159 (13)	0.0814 (10)	
H2A	0.4076	0.5900	−0.0491	0.098*	
H2B	0.2404	0.5789	−0.0350	0.098*	
C3	0.3874 (4)	0.6643 (4)	0.02624 (13)	0.0738 (9)	
H3A	0.3434	0.5930	0.0485	0.089*	
H3B	0.4969	0.6581	0.0326	0.089*	
C5	0.3345 (3)	0.9074 (3)	0.07784 (11)	0.0579 (7)	
C6	0.2855 (3)	1.0463 (3)	0.05824 (10)	0.0532 (7)	
C7	0.2560 (3)	1.0339 (3)	0.00275 (11)	0.0554 (7)	
C8	0.2900 (3)	0.8895 (3)	−0.00953 (11)	0.0556 (7)	
C9	0.3779 (4)	0.8520 (4)	0.12995 (12)	0.0671 (8)	
C10	0.4326 (4)	0.9619 (4)	0.17365 (12)	0.0662 (8)	
C11	0.4400 (5)	1.0161 (5)	0.26432 (12)	0.0886 (11)	
H11A	0.4017	1.1169	0.2603	0.106*	
H11B	0.5497	1.0193	0.2686	0.106*	
C12	0.3840 (5)	0.9433 (5)	0.31076 (13)	0.1027 (13)	
H12A	0.4207	0.9967	0.3422	0.154*	
H12B	0.4199	0.8427	0.3135	0.154*	
H12C	0.2753	0.9436	0.3065	0.154*	
C13	0.2684 (3)	1.1863 (3)	0.08767 (11)	0.0597 (8)	
C14	0.1747 (3)	1.1872 (3)	0.13189 (11)	0.0587 (8)	
C15	0.1881 (4)	1.3048 (4)	0.16630 (14)	0.0828 (10)	
H15A	0.2562	1.3811	0.1622	0.099*	
C16	0.0987 (6)	1.3085 (5)	0.20733 (15)	0.1011 (13)	
H16A	0.1109	1.3859	0.2316	0.121*	
C17	−0.0070 (4)	1.2007 (5)	0.21290 (13)	0.0822 (11)	
C18	−0.0224 (4)	1.0875 (5)	0.17767 (12)	0.0796 (10)	
H18A	−0.0952	1.0146	0.1805	0.096*	
C19	0.0685 (3)	1.0794 (4)	0.13783 (11)	0.0645 (8)	
H19A	0.0579	0.9997	0.1145	0.077*	
C20	−0.1077 (8)	1.2103 (7)	0.2552 (2)	0.164 (2)	
H20A	−0.2045	1.1678	0.2429	0.247*	
H20B	−0.1206	1.3126	0.2644	0.247*	
H20C	−0.0632	1.1567	0.2856	0.247*	
C21	0.1933 (3)	1.1473 (3)	−0.03498 (11)	0.0552 (7)	
C22	0.0754 (4)	1.2406 (4)	−0.02484 (14)	0.0726 (9)	
H22A	0.0359	1.2324	0.0071	0.087*	
C23	0.0156 (4)	1.3444 (4)	−0.06075 (17)	0.0873 (11)	
H23A	−0.0625	1.4061	−0.0528	0.105*	
C24	0.0712 (5)	1.3571 (5)	−0.10828 (18)	0.0975 (13)	
H24A	0.0316	1.4277	−0.1326	0.117*	
C25	0.1858 (5)	1.2647 (5)	−0.11977 (14)	0.0901 (11)	
H25A	0.2229	1.2722	−0.1522	0.108*	
C26	0.2464 (4)	1.1607 (4)	−0.08351 (12)	0.0696 (9)	
H26A	0.3239	1.0988	−0.0918	0.083*	

### Atomic displacement parameters (Å^2^)

**Table:**

	*U*^11^	*U*^22^	*U*^33^	*U*^12^	*U*^13^	*U*^23^
O1	0.170 (3)	0.0612 (18)	0.0835 (16)	0.0110 (17)	−0.0077 (17)	0.0117 (14)
O2	0.1034 (19)	0.091 (2)	0.0803 (16)	−0.0263 (16)	0.0085 (14)	−0.0017 (14)
O3	0.0979 (17)	0.0886 (18)	0.0579 (13)	−0.0101 (13)	0.0045 (12)	−0.0020 (12)
O4	0.1009 (17)	0.0537 (15)	0.0960 (16)	−0.0161 (13)	0.0199 (14)	−0.0042 (12)
N4	0.0605 (14)	0.0468 (16)	0.0676 (15)	0.0063 (11)	0.0018 (12)	−0.0051 (13)
C1	0.069 (2)	0.069 (2)	0.0695 (19)	0.0092 (17)	0.0026 (16)	−0.0146 (17)
C2	0.097 (3)	0.058 (2)	0.089 (2)	−0.0014 (19)	0.013 (2)	−0.0175 (19)
C3	0.075 (2)	0.050 (2)	0.094 (2)	0.0097 (16)	−0.0015 (18)	−0.0107 (17)
C5	0.0598 (17)	0.0507 (18)	0.0615 (17)	0.0002 (14)	0.0002 (14)	−0.0015 (15)
C6	0.0529 (16)	0.0460 (18)	0.0601 (17)	−0.0029 (13)	0.0043 (13)	0.0000 (13)
C7	0.0504 (16)	0.0538 (19)	0.0618 (17)	0.0000 (14)	0.0049 (13)	0.0000 (15)
C8	0.0520 (16)	0.0535 (19)	0.0607 (17)	0.0015 (14)	0.0040 (13)	−0.0046 (15)
C9	0.077 (2)	0.055 (2)	0.068 (2)	0.0061 (17)	0.0021 (16)	0.0057 (17)
C10	0.069 (2)	0.069 (2)	0.0587 (19)	0.0076 (18)	−0.0027 (16)	0.0032 (17)
C11	0.111 (3)	0.091 (3)	0.0593 (19)	−0.009 (2)	−0.010 (2)	−0.0042 (19)
C12	0.140 (4)	0.099 (3)	0.069 (2)	0.000 (3)	0.014 (2)	−0.010 (2)
C13	0.0639 (18)	0.049 (2)	0.0635 (17)	0.0015 (15)	−0.0021 (15)	−0.0010 (15)
C14	0.0654 (18)	0.053 (2)	0.0555 (16)	0.0093 (15)	−0.0005 (15)	−0.0057 (14)
C15	0.097 (3)	0.063 (2)	0.090 (2)	−0.0023 (19)	0.016 (2)	−0.0190 (19)
C16	0.132 (4)	0.085 (3)	0.089 (3)	0.009 (3)	0.024 (3)	−0.031 (2)
C17	0.090 (2)	0.094 (3)	0.064 (2)	0.025 (2)	0.0157 (19)	−0.004 (2)
C18	0.078 (2)	0.097 (3)	0.0652 (19)	0.001 (2)	0.0114 (18)	0.002 (2)
C19	0.0686 (19)	0.065 (2)	0.0590 (17)	0.0005 (17)	0.0047 (15)	−0.0028 (15)
C20	0.196 (6)	0.182 (6)	0.121 (4)	0.035 (5)	0.043 (4)	−0.005 (4)
C21	0.0528 (16)	0.0464 (17)	0.0653 (17)	−0.0040 (13)	0.0015 (14)	0.0011 (14)
C22	0.067 (2)	0.064 (2)	0.086 (2)	0.0044 (17)	0.0057 (18)	0.0089 (18)
C23	0.075 (2)	0.067 (3)	0.116 (3)	0.0088 (19)	−0.005 (2)	0.014 (2)
C24	0.105 (3)	0.074 (3)	0.104 (3)	−0.004 (2)	−0.025 (3)	0.024 (2)
C25	0.114 (3)	0.083 (3)	0.073 (2)	−0.019 (3)	0.006 (2)	0.019 (2)
C26	0.071 (2)	0.068 (2)	0.0695 (19)	−0.0078 (17)	0.0073 (17)	0.0037 (17)

### Geometric parameters (Å, º)

**Table:**

O1—C9	1.211 (4)	C12—H12B	0.9600
O2—C10	1.190 (4)	C12—H12C	0.9600
O3—C10	1.308 (4)	C13—C14	1.482 (4)
O3—C11	1.463 (4)	C14—C15	1.373 (4)
O4—C13	1.218 (3)	C14—C19	1.375 (4)
N4—C8	1.339 (4)	C15—C16	1.389 (5)
N4—C5	1.380 (3)	C15—H15A	0.9300
N4—C3	1.460 (4)	C16—C17	1.370 (6)
C1—C8	1.493 (4)	C16—H16A	0.9300
C1—C2	1.509 (5)	C17—C18	1.356 (5)
C1—H1A	0.9700	C17—C20	1.486 (6)
C1—H1B	0.9700	C18—C19	1.375 (4)
C2—C3	1.521 (4)	C18—H18A	0.9300
C2—H2A	0.9700	C19—H19A	0.9300
C2—H2B	0.9700	C20—H20A	0.9600
C3—H3A	0.9700	C20—H20B	0.9600
C3—H3B	0.9700	C20—H20C	0.9600
C5—C6	1.397 (4)	C21—C26	1.384 (4)
C5—C9	1.433 (4)	C21—C22	1.392 (4)
C6—C7	1.417 (4)	C22—C23	1.373 (5)
C6—C13	1.485 (4)	C22—H22A	0.9300
C7—C8	1.379 (4)	C23—C24	1.370 (5)
C7—C21	1.469 (4)	C23—H23A	0.9300
C9—C10	1.529 (4)	C24—C25	1.374 (6)
C11—C12	1.493 (5)	C24—H24A	0.9300
C11—H11A	0.9700	C25—C26	1.382 (5)
C11—H11B	0.9700	C25—H25A	0.9300
C12—H12A	0.9600	C26—H26A	0.9300
			
C10—O3—C11	116.9 (3)	C11—C12—H12C	109.5
C8—N4—C5	110.2 (2)	H12A—C12—H12C	109.5
C8—N4—C3	114.0 (2)	H12B—C12—H12C	109.5
C5—N4—C3	135.3 (3)	O4—C13—C14	120.8 (3)
C8—C1—C2	103.3 (3)	O4—C13—C6	119.7 (3)
C8—C1—H1A	111.1	C14—C13—C6	119.5 (3)
C2—C1—H1A	111.1	C15—C14—C19	118.8 (3)
C8—C1—H1B	111.1	C15—C14—C13	118.7 (3)
C2—C1—H1B	111.1	C19—C14—C13	122.4 (3)
H1A—C1—H1B	109.1	C14—C15—C16	119.3 (4)
C1—C2—C3	107.6 (3)	C14—C15—H15A	120.4
C1—C2—H2A	110.2	C16—C15—H15A	120.4
C3—C2—H2A	110.2	C17—C16—C15	121.5 (3)
C1—C2—H2B	110.2	C17—C16—H16A	119.3
C3—C2—H2B	110.2	C15—C16—H16A	119.3
H2A—C2—H2B	108.5	C18—C17—C16	118.7 (3)
N4—C3—C2	101.8 (3)	C18—C17—C20	120.3 (4)
N4—C3—H3A	111.4	C16—C17—C20	120.9 (4)
C2—C3—H3A	111.4	C17—C18—C19	120.6 (4)
N4—C3—H3B	111.4	C17—C18—H18A	119.7
C2—C3—H3B	111.4	C19—C18—H18A	119.7
H3A—C3—H3B	109.3	C14—C19—C18	121.1 (3)
N4—C5—C6	106.5 (2)	C14—C19—H19A	119.5
N4—C5—C9	120.4 (3)	C18—C19—H19A	119.5
C6—C5—C9	133.1 (3)	C17—C20—H20A	109.5
C5—C6—C7	107.6 (3)	C17—C20—H20B	109.5
C5—C6—C13	128.6 (3)	H20A—C20—H20B	109.5
C7—C6—C13	123.8 (3)	C17—C20—H20C	109.5
C8—C7—C6	106.4 (3)	H20A—C20—H20C	109.5
C8—C7—C21	125.6 (3)	H20B—C20—H20C	109.5
C6—C7—C21	127.9 (3)	C26—C21—C22	117.3 (3)
N4—C8—C7	109.3 (2)	C26—C21—C7	120.4 (3)
N4—C8—C1	110.0 (3)	C22—C21—C7	122.2 (3)
C7—C8—C1	140.7 (3)	C23—C22—C21	121.8 (3)
O1—C9—C5	123.4 (3)	C23—C22—H22A	119.1
O1—C9—C10	117.5 (3)	C21—C22—H22A	119.1
C5—C9—C10	118.9 (3)	C24—C23—C22	119.9 (4)
O2—C10—O3	125.7 (3)	C24—C23—H23A	120.0
O2—C10—C9	122.3 (3)	C22—C23—H23A	120.0
O3—C10—C9	111.9 (3)	C23—C24—C25	119.6 (4)
O3—C11—C12	106.5 (3)	C23—C24—H24A	120.2
O3—C11—H11A	110.4	C25—C24—H24A	120.2
C12—C11—H11A	110.4	C24—C25—C26	120.5 (4)
O3—C11—H11B	110.4	C24—C25—H25A	119.8
C12—C11—H11B	110.4	C26—C25—H25A	119.8
H11A—C11—H11B	108.6	C25—C26—C21	120.9 (3)
C11—C12—H12A	109.5	C25—C26—H26A	119.6
C11—C12—H12B	109.5	C21—C26—H26A	119.6
H12A—C12—H12B	109.5		
			
C8—C1—C2—C3	−16.0 (4)	O1—C9—C10—O3	−44.2 (4)
C8—N4—C3—C2	−14.4 (3)	C5—C9—C10—O3	141.7 (3)
C5—N4—C3—C2	175.1 (3)	C10—O3—C11—C12	−173.4 (3)
C1—C2—C3—N4	18.3 (3)	C5—C6—C13—O4	127.9 (3)
C8—N4—C5—C6	0.8 (3)	C7—C6—C13—O4	−50.6 (4)
C3—N4—C5—C6	171.6 (3)	C5—C6—C13—C14	−54.4 (4)
C8—N4—C5—C9	179.9 (3)	C7—C6—C13—C14	127.2 (3)
C3—N4—C5—C9	−9.3 (5)	O4—C13—C14—C15	−17.5 (5)
N4—C5—C6—C7	0.1 (3)	C6—C13—C14—C15	164.8 (3)
C9—C5—C6—C7	−178.9 (3)	O4—C13—C14—C19	158.4 (3)
N4—C5—C6—C13	−178.6 (3)	C6—C13—C14—C19	−19.3 (4)
C9—C5—C6—C13	2.5 (5)	C19—C14—C15—C16	2.7 (5)
C5—C6—C7—C8	−0.8 (3)	C13—C14—C15—C16	178.7 (3)
C13—C6—C7—C8	177.9 (3)	C14—C15—C16—C17	−2.8 (6)
C5—C6—C7—C21	175.8 (3)	C15—C16—C17—C18	0.8 (6)
C13—C6—C7—C21	−5.4 (5)	C15—C16—C17—C20	−176.6 (4)
C5—N4—C8—C7	−1.4 (3)	C16—C17—C18—C19	1.4 (5)
C3—N4—C8—C7	−174.3 (2)	C20—C17—C18—C19	178.8 (4)
C5—N4—C8—C1	177.6 (2)	C15—C14—C19—C18	−0.6 (5)
C3—N4—C8—C1	4.7 (3)	C13—C14—C19—C18	−176.5 (3)
C6—C7—C8—N4	1.3 (3)	C17—C18—C19—C14	−1.5 (5)
C21—C7—C8—N4	−175.4 (2)	C8—C7—C21—C26	−41.7 (4)
C6—C7—C8—C1	−177.1 (4)	C6—C7—C21—C26	142.2 (3)
C21—C7—C8—C1	6.1 (6)	C8—C7—C21—C22	135.5 (3)
C2—C1—C8—N4	7.4 (3)	C6—C7—C21—C22	−40.6 (5)
C2—C1—C8—C7	−174.2 (4)	C26—C21—C22—C23	−1.5 (5)
N4—C5—C9—O1	−15.8 (5)	C7—C21—C22—C23	−178.8 (3)
C6—C5—C9—O1	163.1 (3)	C21—C22—C23—C24	0.7 (6)
N4—C5—C9—C10	158.0 (3)	C22—C23—C24—C25	0.5 (6)
C6—C5—C9—C10	−23.2 (5)	C23—C24—C25—C26	−0.8 (6)
C11—O3—C10—O2	−0.7 (5)	C24—C25—C26—C21	−0.1 (5)
C11—O3—C10—C9	174.8 (3)	C22—C21—C26—C25	1.2 (5)
O1—C9—C10—O2	131.5 (4)	C7—C21—C26—C25	178.5 (3)
C5—C9—C10—O2	−42.7 (5)		

### Hydrogen-bond geometry (Å, º)

Cg4 is the centroid of the C21–C26 phenyl ring.

**Table:**

*D*—H···*A*	*D*—H	H···*A*	*D*···*A*	*D*—H···*A*
C20—H20*B*···O3^i^	0.96	2.64	3.268 (6)	123
C12—H12*B*···O2^ii^	0.96	2.63	3.579 (5)	170
C12—H12*A*···O1^iii^	0.96	2.66	3.376 (5)	132
C2—H2*A*···O4^iv^	0.97	2.70	3.391 (5)	128
C19—H19···*Cg*4^v^	0.93	2.96	3.769 (5)	147

Symmetry codes: (i) −*x*, *y*+1/2, −*z*+1/2; (ii) −*x*+1, *y*−1/2, −*z*+1/2; (iii) −*x*+1, *y*+1/2, −*z*+1/2; (iv) −*x*+1, −*y*+2, −*z*; (v) −*x*, −*y*, −*z*.
